# Olanzapine in pregnancy and breastfeeding: a review of data from global safety surveillance

**DOI:** 10.1186/2050-6511-14-38

**Published:** 2013-08-01

**Authors:** Elizabeth Brunner, Deborah M Falk, Meghan Jones, Debashish K Dey, Chetan Chinmaya Shatapathy

**Affiliations:** 1 Eli Lilly and Company, Lilly Corporate Center, Indianapolis, Indiana 46285, USA

**Keywords:** Olanzapine, Breastfeeding, Pregnancy

## Abstract

**Background:**

Olanzapine use has been reported during pregnancy and breastfeeding, but there are no controlled clinical trials assessing the safety of olanzapine exposure to infants and fetuses. The purpose of this report was to review and analyze prospective post-marketing cases of pregnancy and breastfeeding with olanzapine, in order to guide clinicians and women on the use of olanzapine therapy during pregnancy and/or breastfeeding.

**Methods:**

A worldwide safety database maintained by Eli Lilly and Company was searched for all spontaneous-reported data regarding olanzapine use during pregnancy and/or breastfeeding. Cases reported prior to pregnancy outcome were considered to be prospective, and follow-up was pursued after the delivery date to assess outcome.

**Results:**

Outcome data were available for 610 prospectively identified pregnancies during which olanzapine was used. The majority of women had normal births (66%), although premature births were reported in 9.8% and perinatal conditions in 8% of the pregnancies. A total of 102 pregnancies reported olanzapine treatment during breastfeeding. In these infants, the most commonly reported adverse events were somnolence (3.9%), irritability (2%), tremor (2%), and insomnia (2%), although the majority of pregnancies reported no adverse events (82.3%).

**Conclusions:**

The frequency of fetal outcomes in these prospectively identified pregnancies exposed to olanzapine did not differ from rates of outcomes reported in the general population. These data may be useful to help guide clinicians and women decide to continue, or discontinue, olanzapine therapy during pregnancy and/or breastfeeding, but should be considered within the limitations associated with spontaneously reported data. Women should notify their clinicians if they become pregnant or intend to become pregnant while being treated with olanzapine. Because of limited experience in humans, olanzapine should be used in pregnancy only when potential benefit justifies potential risk to the fetus. Olanzapine should only be considered during breastfeeding when the potential benefit justifies the potential risk to the infant.

## Background

Women with psychiatric conditions may become pregnant, and motherhood is common in such women: in one sample, 63% of women with psychotic disorders were mothers [1]. Women with psychotic disorders who are pregnant or breastfeeding are often treated with antipsychotics [2]. Due to ethical constraints restricting inclusion of pregnant and breastfeeding women in clinical trials, there is a paucity of data available on the use of antipsychotic drugs in this population. The current literature on antipsychotic use during pregnancy and breastfeeding stems from case reports and large, uncontrolled reports of prospective and retrospective data [3,4], making it difficult to draw conclusions concerning the safety of these medications for the mother and child. Adverse outcomes during pregnancy, delivery, post-natal care as well as birth defects, and perinatal complications have been reported in patients treated with antipsychotics [4].

Women with schizophrenia and bipolar disorder have a greater likelihood of complications, including placental abnormalities; hemorrhaging; fetal distress; congenital anomalies, such as cardiovascular defects; and neonatal complications [5]. Additionally, cessation of antipsychotic treatment for women with psychotic disorders may increase the risk of relapse, which in turn could lead to poor pre- and post-natal care as well as obstetric-related adverse events [4]. Therefore, clinicians and women must carefully weigh the benefits and the risks of remaining on or terminating antipsychotic treatment.

Olanzapine has been shown to be effective for treating the symptoms of schizophrenia and bipolar disorder in adults and schizophrenia and acute manic or mixed episodes associated with bipolar disorder in adolescents [6-10]. There have been reports about the safety of olanzapine in the fetus and infant, with some cases reporting normal births without complications and others reporting adverse outcomes, such as differences in birth weight [11] and neural development [2]. In preclinical trials, prenatal treatment with olanzapine did not disrupt spatial memory and short-term memory in rats, whereas other antipsychotics did [12].

In the absence of adequate and well-controlled clinical trials, spontaneous post-marketing adverse event data provides information on the safety of treatment with antipsychotic medications during pregnancy and breastfeeding. Spontaneous post-marketing data typically involve a much greater number of exposures to a much broader population of patients compared with data from clinical trials. This is particularly true in this population (women who are pregnant or breastfeeding), since pregnant women are excluded from clinical trials. However, this should be considered within the limitations associated with spontaneously reported data, which may sometimes under represent the true incidence of events, as not all patients or clinicians report pregnancies and their outcomes to Eli Lilly and Company. Here we present prospectively collected data from Eli Lilly and Company’s safety database including spontaneous reports, clinical trial cases, and post-marketing observational study reports, regarding the use of olanzapine during pregnancy and/or breastfeeding.

## Methods

### Database

Eli Lilly and Company (Lilly) has maintained a world-wide safety database of all adverse events reported to Lilly relative to treatment with products marketed by Lilly since 1983. This database consists of all spontaneous adverse events—regardless of severity—reported in patients treated with olanzapine (including data from published literature and regulatory agency reports), and serious adverse event reports from clinical trials and post-marketing studies. Additionally, the database contains reports of olanzapine use during pregnancy and/or breastfeeding, even if no adverse outcome was reported. Cases are entered into the database regardless of the reporter type (e.g., healthcare provider, patient), concomitant medications or medical co-morbidities, or consideration of the potential relationship between olanzapine treatment and the outcome. In the cases that were reported from clinical trials (the minority of cases), the institutional review boards approved the protocols for all these trials and studies were conducted in accordance with ethical principles of Good Clinical Practice and the Declaration of Helsinki and its guidelines. This review was a retrospective analysis of data from the Lilly Safety Database (the Lilly Safety Database is the global database application used for the collection, storage, and reporting of adverse events to regulatory agencies, investigators, and internal departments). The information that was prospectively collected is derived from spontaneously reported pregnancies. In all cases, the initial contact to Lilly is made by the reporter. Lilly then sends a letter and a questionnaire requesting additional information. When information is not provided directly by the patient, Lilly also requests the patient’s consent for release of medical information. Authorizations were not possible to obtain from patients who did not provide any follow-up information. Since the population in this review consists of pregnancies with prospective follow-up data, subjects without any follow-up information were not included in this review. Ethical or Institutional Review Board approval is not mandated (as opposed to prospectively collected data for study purposes from clinical trial participants).

Reports of olanzapine exposure during pregnancy were categorized based on when the report was received relative to the report of the pregnancy outcome. For prospective pregnancy cases (in which the olanzapine exposure was reported before the report of the pregnancy outcome), each reporter was contacted after the expected delivery date for outcomes. Retrospective reports were not used to calculate frequency of pregnancy outcomes for comparison with the general population, since not all olanzapine exposures resulting in pregnancy, nor all outcomes of pregnancy, are reported.

### Definitions

An event represents a clinical sign, symptom, or syndrome reported for a single reported outcome; therefore, more than one event may occur in a patient or the patient’s child. Normal birth was defined as birth between 37 and 42 weeks of gestation, or at an undefined gestation time with no reported abnormalities; premature birth was defined as birth before 37 weeks of gestation; and post-term birth was defined as birth after 42 weeks of gestation.

As per the World Health Organization (WHO), ‘Congenital anomalies, also known as birth defects, are structural or functional abnormalities, including metabolic disorders, which are present from birth. Congenital anomalies are a diverse group of disorders of prenatal origin which can be caused by single gene defects, chromosomal disorders, multifactorial inheritance, environmental teratogens and micronutrient deficiencies [13].

Perinatal condition was defined as an adverse event occurring within 7 days of birth, and post-perinatal condition was defined as an adverse event occurring any time after 7 days post-birth. Elective termination was defined as a planned abortion with no anomalies; therapeutic abortion as a planned abortion due to medical reasons; and a spontaneous abortion as a failure of embryonic development, fetal death in utero, or expulsion of any of all or part of the product of conception before the 20th week of gestation or a fetal weight of <500 grams. Stillbirth was defined as death of the fetus at any time after the 20th week of gestation, with no breathing or other evidence of life after birth, and may also be referred to as intrauterine death. Neonates born at 37 to 42 weeks’ gestation, or at an unspecified gestation, with no reported adverse events were considered full term. Neonates born before 37 weeks’ gestation were considered premature, while neonates born after 42 weeks of gestation were considered post-term.

After birth, neonates were classified as normal if there were no issues noted. A congenital anomaly was defined as a tissue malformation seen in a neonate, born at 37 to 42 weeks’ gestation, or at an unspecified gestation, with a congenital anomaly noted at birth, or a report of a therapeutic abortion due to congenital anomalies in the fetus. A perinatal condition was defined as an adverse event (not considered to be a congenital anomaly) that occurred within 7 days of birth in neonates born at 37 to 42 weeks’ gestation or at an unspecified gestation. A post-perinatal condition was defined as an adverse event (not considered to be a congenital anomaly) that occurred after 7 days of birth in neonates born at 37 to 42 weeks’ gestation or at an unspecified gestation age.

### Analysis

The safety database was searched for all reports of pregnancy and breastfeeding in temporal association with treatment with olanzapine occurring from first (10 September 1986) human dose in a clinical trial through 31 December 2010. Pregnancy outcomes, as well as trimester of olanzapine exposure, were analyzed through 31 December 2010. Qualitative comparisons were made between this dataset and historic reports from the general population on rates of outcomes of and during pregnancy, delivery, and fetal outcomes (see Table 1) [14-24].

**Table 1:** **Fetal outcomes in prospectively identified olanzapine-exposed pregnancies, compared to rates in the general population**^**†**^

**Fetal outcome**	**Outcome reported (%) (N=610)**	**Historic control rate in general population (%)**
Spontaneous abortion	57 (9.3%)^g^	10% to 20% [19,20]
Ectopic pregnancy	3 (0.5%)	1.3% to 2.1% [21-23]
Normal birth^a^	401 (65.7%)	61% to 64% [24]
Premature^b^	60 (9.8%)	12.8% [25]
Post-term^c^	5 (0.8%)	5.6% [25]
Stillbirth	5 (0.8%)^h^	0.5% to 1.1%^i^[26,27]
Congenital anomaly^d^	27 (4.4%)	3.0% to 5.0% [28,29]
Perinatal condition^e^	49 (8.0%)	^j^
Post-perinatal condition^f^	3 (0.5%)	^j^

^a^ Includes neonates born at 37–42 weeks’ gestation, or at an unspecified gestation.

^b^ Includes neonates born <37 weeks’ gestation or reported as “premature.”

^c^ Includes neonates born >42 weeks or reported as “post-term.”

^d^ Includes neonates born at 37–42 weeks’ gestation or at an unspecified gestation with a congenital abnormality (resulting from abnormal tissue formation) at birth, and reports of therapeutic abortions due to congenital abnormalities in the fetus.

^e^ Includes neonates born at 37–42 weeks’ gestation or at an unspecified gestation with adverse event ≤7 days of birth.

^f^ Includes neonates born at 37–42 weeks’ gestation or at an unspecified gestation with an adverse event >7 days after birth.

^g^ Includes one report of a congenital anomaly in a 13-week aborted fetus.

^h^ Includes one report of a normal fetus who died when the mother committed suicide at 8 months’ gestation.

^i^ Indicates range when stratified by race/ethnicity.

^j^ Due to the specific definitions (gestation and adverse events in a timeframe after birth), historical population rates are not available.

^**†**^ Clinical trial and spontaneous reports from the Lilly worldwide safety database (First human dose through 31 December 2010).

## Results

### Pregnancy outcomes

Through 31 December 2010, there were 610 prospectively identified pregnancies with an available outcome reported and included in this analysis. In addition, 73 cases reported an elective termination without a fetal anomaly, and were not included in the analysis. Maternal oral olanzapine dose was reported in 535 of 610 (87.7%) pregnancies and oral olanzapine doses ranged from 0.6 mg/day to 35.0 mg/day, with a mean dose of 10.3 mg/day. Intramuscular injections were reported in several cases (<1%) with reported maternal doses within the labeled dose range.

Of the 610 prospectively identified pregnancies exposed to olanzapine with an available outcome, there were 401 (66%) normal births, 60 (9.8%) premature births, 57 (9.3%) spontaneous abortions, 49 (8%) perinatal conditions, 27 (4.4%) congenital anomalies, and 16 (2.6%) other (post-perinatal condition, ectopic pregnancy, post-term birth, and stillbirth). There did not appear to be an increased risk of spontaneous abortion, ectopic pregnancy, stillbirth, premature or post-term birth, or congenital anomalies in pregnant women treated with olanzapine compared with historic control rates in the general population (Table 1). Given the well-known limitations of the data under review, the findings need to be interpreted with caution.

The timing of exposure to olanzapine during pregnancy was reported in 594 (97.4%) of the prospectively reported cases. Of these, the majority reported olanzapine exposure either during all three trimesters 263 (44.3%) or in the first trimester only 187 (31.5%). Approximately 47.1% (189/401) of women experiencing normal births were exposed to olanzapine during all three trimesters. The majority of women who experienced spontaneous abortions were exposed to olanzapine during the first trimester only (50/57, 87.7%). Approximately half of the women who experienced premature (27/60, 45%) or post-mature births (3/5, 60%) were treated with olanzapine throughout pregnancy. Among women who experienced perinatal conditions, 63.3% (31/49) were exposed to olanzapine for all three trimesters and 6.1% (3/49) were exposed to olanzapine during the third trimester only (Table 2). Forty-three percent of women reported continuation of treatment with olanzapine during all three trimesters of their pregnancy (Table 2). In patients who continued olanzapine treatment throughout all three trimesters, 71.9% had normal births. Exposure during only the first trimester of pregnancy was reported in 30.7% of pregnancies.

**Table 2:** **Trimester of olanzapine exposure in prospectively identified pregnancies by outcome from Lilly Worldwide Safety Database**^**†**^

	**Outcomes reported by trimester(s) of exposure to Olanzapine (n)**								
**Pregnancy outcome**	**1**^**st **^**only**	**2**^**nd **^**only**	**3**^**rd **^**only**	**1**^**st **^**& 2**^**nd**^	**1 & 3**^**rd**^	**2**^**nd **^**& 3**^**rd**^	**All**	**Unknown**	**Total**
**Normal birth**^**a**^	109	14	18	37	7	21	189	6	**401**
Full-term	96	12	17	33	6	20	169	4	**357**
Unknown gestation	13	2	1	4	1	1	20	2	**44**
**Spontaneous abortion**^**a**^	50	1	0	4	0	0	0	2	**57**^**g**^
**Ectopic pregnancy**	3	0	0	0	0	0	0	0	**3**
**Premature**^**b**^	11	1	2	4	1	11	27	3	**60**
Normal	7	1	0	3	1	8	18	3	**41**
Congenital anomaly	2	0	0	0	0	0	2	0	**4**
Perinatal condition	2	0	2	1	0	3	7	0	**15**
**Post-term**^**c**^	0	0	2	0	0	0	3	0	**5**
Normal	0	0	2	0	0	0	1	0	**3**
Perinatal condition	0	0	0	0	0	0	2	0	**2**
**Stillbirth**	0	0	0	0	0	1	4	0	**5**^**h**^
**Congenital anomaly**^**d**^	3	1	2	4	0	7	8	2	**27**
Full-term	2	1	2	1	0	4	8	0	**18**
Unknown gestation	0	0	0	2	0	3	0	2	**7**
Therapeutic abortion	1	0	0	1	0	0	0	0	**2**
**Perinatal condition**^**e**^	9	0	3	2	0	1	31	3	**49**
Full-term	8	0	3	1	0	1	28	3	**44**
Unknown gestation	1	0	0	1	0	0	3	0	**5**
**Post-perinatal condition**^**f**^	2	0	0	0	0	0	1	0	**3**
Full-term	2	0	0	0	0	0	1	0	**3**
**Totals**	**187**	**17**	**27**	**51**	**8**	**41**	**263**	**16**	**610**

^a^ Includes neonates born at 37–42 weeks’ gestation, or at an unspecified gestation.

^b^ Includes neonates born <37 weeks’ gestation or reported as “premature.”

^c^ Includes neonates born >42 weeks’ or reported as “post-term.”

^d^ Includes neonates born at 37–42 weeks’ gestation or at an unspecified gestation with a congenital abnormality (resulting from abnormal tissue formation) at birth, and reports of therapeutic abortions due to congenital abnormalities in the fetus.

^e^ Includes neonates born at 37–42 weeks’ gestation or at an unspecified gestation with an adverse event ≤7 days after birth.

^f^ Includes neonates born at 37–42 weeks’ gestation or at an unspecified gestation with an adverse event >7 days after birth.

^g^ Includes one report of a congenital anomaly in a 13-week aborted fetus.

^h^ Includes one normal fetus who died when the mother committed suicide at 8 months’ gestation.

^**†**^ First Human Dose through 31 December 2010 (Data on file).

Of the prospectively identified pregnancies with an available reported outcome, 27 (4.4%) reported congenital anomalies; this risk did not appear to be greater in the population being treated with olanzapine compared with the general population (Table 1).

### Breastfeeding

In women being treated with olanzapine while breastfeeding, from spontaneous reports, clinical trial cases, and post-marketing observational study reports, (N=102), 62 pregnancies included olanzapine dose information: doses ranged from 2.5 to 20.0 mg/day, with a mean dose of 7.4 mg/day. All reported an oral dose form. Duration of olanzapine exposure during breastfeeding was reported in 30 pregnancies, and ranged from 2 days to 13 months, with a mean exposure of 74 days, and a median exposure of 30 days. In a study in lactating, healthy women taking oral olanzapine, olanzapine was excreted in breast milk. Mean infant exposure (mg/kg) at steady state was estimated to be 1.8% of the maternal olanzapine dose (mg/kg) [25]. In the olanzapine safety database, there were no adverse events reported in the neonate/infant in the majority (82.3%) of pregnancies that reported breastfeeding during olanzapine treatment. A total of 15.6% of the pregnancies reported an adverse event in the neonate or infant in temporal association with breastfeeding. The most commonly reported adverse events included somnolence (3.9%), irritability (2%), tremor (2%), and insomnia (2%). Outcomes of these events noted in the neonate/infant were reported as recovered/recovering in 40% of the events, as not recovered in 24% of the events, and as unknown in 36% of the events.

## Discussion

Through 31 December 2010, it is estimated that more than 33 million patients were treated with olanzapine. Based on the analysis of prospectively reported pregnancy where there was an outcome available, 66% of the outcomes of women treated with olanzapine at any time during pregnancy were normal. This rate of normal birth outcomes is comparable to that of the general population, which ranges from 61% to 64% [19]. Premature births (9.8%) and spontaneous abortion (9.3%) rates in this dataset were comparable to those of the general population (12.8% [20] and 10% to 20% [14,15], respectively).

Prospective reports are subject to fewer reporting biases compared with retrospective reports [26]. Women whose children have major birth defects (abnormality that can affect the structure or function of an organ) are more likely to report the outcome, compared with women who have healthy babies [27]. Prospectively collected information provides more details about a case and helps to provide a risk estimate [28], whereas retrospective reports may be susceptible to systematic recall bias and underestimation of exposure to maternal psychiatric illness and non-psychotropic agents [29], and cannot be used in the calculation of outcome rates [30]. In addition, prospective cohorts are able to include specific variables, and the data can be collected with more reliability and accuracy [31], thus providing an opportunity to obtain follow-up information, and a more accurate ascertainment of exposure during pregnancy and in the perinatal period. Therefore, in this population, prospective reports were used to calculate frequency of pregnancy outcomes for comparison to the general population.

The risk of unfavorable outcomes in neonates and infants whose mothers were treated with olanzapine during pregnancy in this dataset did not appear to differ from that of the general population. This information is complementary to the findings of a previous prospective study of pregnancy outcomes in patients treated with atypical antipsychotics. The defects in the infant of the olanzapine-treated patient included the midline defects of cleft lip, encephalocele, and aqueductal stenosis. Yet all these events occurred in one child of an olanzapine-treated mother, compared with none in the other treatment groups [2]. Given the well-known limitations of the data under review, the findings need to be interpreted with caution.

Other congenital defects, including meningocele/ankyloblepharon [32], hip dysplasia [33], acheiria [34], and atrioventricular canal defect/unilateral clubfoot [35], have been reported in infants exposed to olanzapine in utero.

In a postmarketing safety database assessment of 68 prospective pregnancies, spontaneously reported with a known outcome in women who received risperidone [3], 37 pregnancies reported a normal outcome. Organ malformations were reported in 3.8% of the reports and spontaneous abortions in 16.9% of the reports. The denominator used was subtracting the number of induced abortions (predominantly undertaken for nonmedical reasons) from the total 68 reports. In utero exposure to risperidone does not appear to increase the risk of spontaneous abortions, structural malformations, and fetal teratogenic risk above that of the general population.

In a prospective, controlled cohort study of 215 pregnancies in women exposed to haloperidol or penfluridol [36], rates of major abnormalities were compared to a control group of pregnancies (N=631) exposed to non-teratogenic agents reported to a European counseling center. Compared to the controlled group, the women treated with antipsychotics were older; and a significantly higher proportion smoked 5 or more cigarettes a day. No differences were noted in the number of previous miscarriages, number of pregnancies, history of elective terminations, or gestational age at first contact. No differences were observed between the antipsychotic-exposed group and the control group in the rates of major malformations (3.4% vs. 3.8% respectively) or in the rates of major malformations in live births with first trimester exposure (3.1% vs. 3.8% respectively). There were statistically significant differences observed between the antipsychotic-exposed group and the control group in the rates of delivery (81.9% vs. 90.3% respectively), elective termination of pregnancy (8.8% vs. 3.8% respectively), preterm birth (13.9% vs. 6.9% respectively), Cesarean section (25.5% vs. 16.3% respectively), and in the median gestational age [interquartile range] at delivery (40 [38-40] vs. 40 [39-41] weeks respectively), median birthweight [interquartile range] (3155 [2800–3500] vs. 3370 [3030–3700] g respectively), and median birthweight [interquartile range] of full-term infants (3250 [3000–3590] vs. 3415 [3140–3750] g respectively).

In this dataset, 66% of births were classified as normal births; premature births occurred in 9.8% of pregnancies. This is not consistent with the findings of Newham et al. [37], who found higher rates of premature births in infants whose mothers were treated with typical antipsychotics compared with a non-medicated reference group, although there was no significant difference between the atypical antipsychotic and the reference groups. This analysis, as ours, did not control for concomitant mediations or other potential confounders, which may have potentially affected gestational age.

Although this analysis did not examine birth weight, previous studies have found higher rates of low birth weight (31%) and neonatal intensive care unit admission (31%) reported in neonates whose mothers were treated with olanzapine during pregnancy compared with neonates of mothers who were treated with other atypical antipsychotics [11], although the differences were not statistically significant. Another study found that infants born to mothers who were treated with olanzapine or clozapine during pregnancy had significantly higher birth weights compared with infants of mothers who were treated with typical antipsychotics [37].

One study examining placental passage (defined as the ratio of umbilical cord to maternal plasma concentration) of antipsychotics found that olanzapine concentrations in the placenta were 72% of the mother’s, which was significantly greater compared with concentrations of haloperidol, quetiapine, and risperidone [11]. However, another study reported the rate of placental passage of olanzapine to be much lower (17%) [38]. Potential fetal exposure to olanzapine should be taken into consideration when weighing the benefits and risks of remaining on olanzapine therapy.

Infant exposure to olanzapine via breast milk of olanzapine-treated mothers was lower than exposure in utero. Adverse events were reported in 15.6% of infants who were exposed to olanzapine through breast milk, and the most common events included somnolence, irritability, tremor, and insomnia. This differs from several studies of olanzapine in which either no adverse events were noted [39-43], or the types of adverse events (e.g., respiratory difficulties and hypotonia) were different [44]. The safety profile of olanzapine is based on exposures to the drug in adults and adolescents, the safety profile of exposure through uterus, during early and late developmental stages or via breast milk is likely to differ. Gilad et al. [44] found that the rate of adverse events in olanzapine-exposed breastfed infants were not statistically significantly different from infants not exposed to olanzapine.

The information reviewed in the current paper was voluntarily reported by (or on behalf of) the patient (spontaneous adverse event reports). There are several, well-known limitations to spontaneously reported data. Spontaneous reporting of adverse events may be highly variable [45]: clinicians may be less likely to report adverse events that are not serious or have been seen in the general population; this may skew the data toward more serious adverse events. Similarly, the information collected may lack details in particular key information, such as concurrent treatments, relevant medical history, and long-term follow-up of infant developmental outcomes. The data reported here included all reported cases regardless of concomitant medications, relevant medical co-morbidities, and potential causal relationship to olanzapine exposure. Since this is not a clinical trial, data are not collected like they typically are in a clinical trial or in a prospective observational study where there is a defined protocol. The follow-up rate cannot be calculated from spontaneously reported data as there is no pre-defined duration of follow-up for individual patient reports. Moreover, due to the nature of spontaneous reporting, and given the process of soliciting and obtaining follow-up information, a follow-up rate often does not provide an informative assessment of the completeness and accuracy of the follow-up. Although follow-up information may have been received, often the most relevant follow-up is the outcome of pregnancy/breastfeeding, which may not always be available. In addition, it is not possible to confirm the exact trimester in which the patients were exposed to olanzapine from spontaneously reported data. In summary, spontaneous data alone are not adequate to make definitive conclusions regarding the potential risk of adverse events following exposure to olanzapine.

Based on reports from the Adverse Event Reporting System (AERS), the Food and Drug Administration’s (FDA) computerized information database designed to monitor for new adverse events and support post-marketing safety surveillance for approved drugs, various major regulatory agencies recently updated the prescribing information label language of all antipsychotics regarding use during pregnancy, informing of the potential risk of abnormal muscle movements (extrapyramidal signs) and/or withdrawal symptoms in neonates whose mothers were treated with antipsychotics during the third trimester of pregnancy [46]. It is important to assess the risks and benefits of treating women who are pregnant or breastfeeding with antipsychotics, and weigh these against possible risks of anomalies and developmental problems to the fetus and infant.

## Conclusions

There are no controlled studies for the use of olanzapine therapy in pregnant women or in women who are breastfeeding. Given the well-known limitations of data under review, the findings need to be interpreted with caution. The relative risks and benefits of olanzapine treatment during pregnancy and/or breastfeeding should be carefully weighed by the clinician and the patient on a case-by-case basis. Because of limited experience in humans, olanzapine should be used in pregnancy only when potential benefit justifies potential risk to the fetus [25]. Olanzapine should only be considered during breastfeeding when the potential benefit justifies the potential risk to the infant. Women should be advised to notify their clinician if they become pregnant or intend to become pregnant during treatment with olanzapine. Presently, data are not sufficient to make definitive conclusions regarding the safety of olanzapine therapy during pregnancy and/or breastfeeding. However, acknowledging the limitations of the existing data, our review found that the frequency of fetal outcomes in prospectively identified pregnancies exposed to olanzapine did not differ from rates of outcomes reported in the general population. In the absence of data from clinical trials, the current analysis of post-marketing data attempts to provide greater information about the safety of olanzapine exposure during pregnancy and/or breastfeeding.

## Competing interests

All authors are employees and stockholders of Eli Lilly and Company.

## Authors’ contributions

All authors (EB, DF, MJ, DD, and CS) have contributed to the conception and planning of the work that led to the manuscript, analysis and interpretation of the data, and drafting and critical revision of the manuscript for intellectual content. All authors read and approved the final manuscript.

## Pre-publication history

The pre-publication history for this paper can be accessed here:

http://www.biomedcentral.com/2050-6511/14/38/prepub
